# Proof-of-Principle Demonstration of Direct Metabolic Imaging Following Myocardial Infarction Using Hyperpolarized 13C CMR

**DOI:** 10.1016/j.jcmg.2020.12.023

**Published:** 2021-06

**Authors:** Andrew Apps, Justin Y.C. Lau, Jack J.J.J. Miller, Andrew Tyler, Liam A.J. Young, Andrew J.M. Lewis, Gareth Barnes, Claire Trumper, Stefan Neubauer, Oliver J. Rider, Damian J. Tyler

Although ischemic heart disease is a major contributor to global disease burden, there remains scope to improve diagnosis, risk stratification, and management of myocardial ischemia. The recent ISCHEMIA (International Study of Comparative Health Effectiveness With Medical and Invasive Approaches) trial showed that after an average follow-up of 3.2 years, invasive therapy did not reduce major adverse cardiac events compared with optimal medical therapy in patients with stable ischemic heart disease (1). The presence of ischemia invariably leads to alterations in the balance between aerobic and anaerobic metabolism, and therefore, noninvasive detection of these metabolic alterations may lead to improvements in patient care pathways. Although current cardiac magnetic resonance (CMR) techniques are able to assess altered perfusion and scar burden, they cannot directly measure metabolic alterations. In addition, whereas positron emission tomography with ^18^F-fluorodeoxyglucose allows assessment of glucose uptake, it is unable to report on the metabolic fate of glucose beyond its initial phosphorylation by hexokinase, and so a new approach is required.

**Figure 1:**
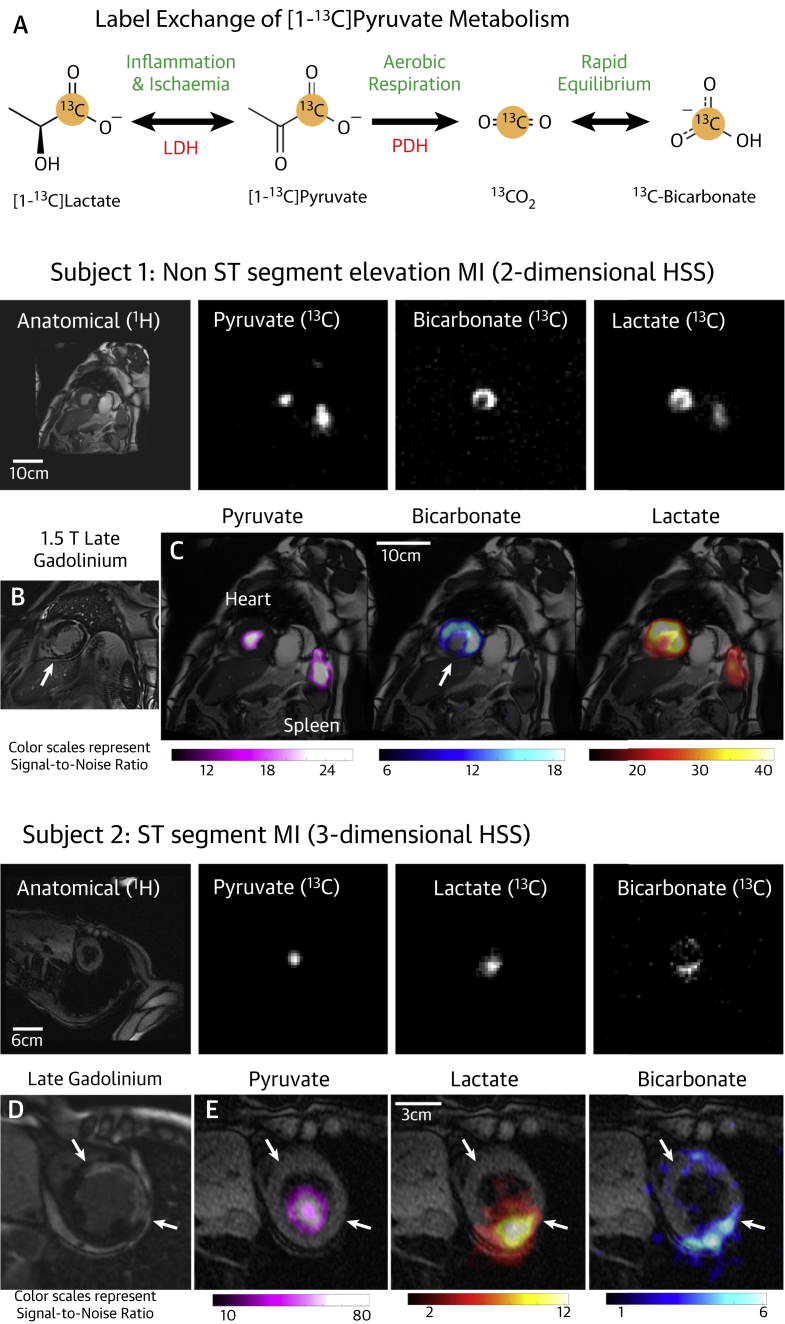
Proof-of-Principle Metabolic Images Acquired From the Ischemic Heart Using Hyperpolarized ^13^C CMR **(A)** Schematic representation of metabolic pathways observable following injection of hyperpolarized [1-^13^C]pyruvate. **(B and C)** Representative late gadolinium/metabolic images acquired from Subject #1. **(D and E)** Representative late gadolinium/metabolic images acquired from Subject #2. CMR = cardiac magnetic resonance; HSS = hybrid-shot spiral; LDH = lactate dehydrogenase; PDH = pyruvate dehydrogenase.

The fate of glucose metabolism after glycolysis depends on the prevailing metabolic conditions and thus has the potential to be used diagnostically, with the equilibrium between pyruvate dehydrogenase (PDH) activity and lactate dehydrogenase (LDH) activity indicating the balance between aerobic and anaerobic metabolism (2). The recently demonstrated technique of hyperpolarized cardiac magnetic resonance (hp-CMR) offers the ability to noninvasively monitor PDH and LDH activity (3), and may provide the potential for direct imaging of metabolism in the ischemic heart (Figure 1A). Whereas this potential has been established in animal models (4,5), we present here the first hp-CMR images of pathological human myocardial metabolism in ischemic heart disease.

Studies were approved by the National Research Ethics Committee (17/WM/0200). Hyperpolarized [1-^13^C]pyruvate was prepared in a GE SPINlab hyperpolarizer (GE Healthcare, Chicago, Illinois) and administered intravenously (0.1 mmol/kg) (3). Hyperpolarized ^13^C images were acquired on a Siemens 3T Tim Trio scanner (Siemens Healthineers, Erlangen, Germany) using a cardiac-gated sequence consisting of interleaved spectral-spatial excitations of pyruvate, lactate, and bicarbonate resonances followed by a hybrid-shot spiral (HSS) readout (6). A 2-dimensional implementation of HSS was used in case 1 and encoded 3 short-axis slices (basal, mid, apical) of 20-mm thickness per heartbeat with nominal 10-mm in-plane resolution (flip angles: pyruvate 12°; lactate/bicarbonate 60°). Imaging was performed over an end-expiration breath-hold started 22 s after injection; all 3 slices were encoded each heartbeat for 1 metabolite, and the 3 metabolites were acquired over 3 subsequent heartbeats in the order pyruvate, bicarbonate, and lactate. Three interleaves were used to acquire the presented data, requiring 9 heartbeats in total. For case 2, a 3-dimensional implementation of HSS was used and encoded a 384 × 384 × 120 mm^3^ volume with nominal 6-mm in-plane resolution and 3 excitations per heartbeat (flip angles: pyruvate 6°; lactate/bicarbonate 30°) and 12 excitations per volume. As for the 2-dimensional case, 3 interleaves were used to acquire the presented data, requiring 36 heartbeats in total.

Case 1: A 67-year-old man with type 2 diabetes presented with chest pain, non–ST-segment elevation myocardial infarction (cardiac troponin I 44 ng/l), and electrocardiographic evidence of anterolateral territory ischemia. Coronary angiography revealed disease of the distal left main and proximal left anterior descending coronary arteries with angiographic appearances consistent with a chronic total occlusion of the right coronary artery, which was dominant. CMR and late gadolinium enhancement imaging were undertaken to assess viability and inform revascularization. This demonstrated 2 separate areas of infarction: subendocardial infarction (25% to 50%, intermediate viability, presumed acute) of the mid- and apical anterior and anterolateral walls (4 of 17 segments), and transmural (75% to 100%, nonviable, presumed old) infarction of the inferior septum (2 of 17 segments) (Figure 1B). Hyperpolarized [1-^13^C]pyruvate imaging (Figure 1C) was undertaken 5 days following the onset of chest pain and showed an absence of ^13^C-bicarbonate and [1-^13^C]lactate signals in the nonviable inferior septum, but ^13^C-bicarbonate and [1-^13^C]lactate signals were seen in the anterior wall in the region of the subendocardial infarction, demonstrating ongoing oxidative metabolism in the recently infarcted anterior wall.

Case 2: A 76-year-old woman presented 24 h after a severe episode of chest pain to a regional hospital on the island of Jersey. By this time, anterior Q waves were seen on the electrocardiogram; however, pain persisted and ST-segment elevation was still apparent, so the patient was treated with intravenous thrombolytic therapy and flown to our center with the capability for primary coronary intervention for ongoing management. On arrival, the patient was stable without symptoms; echocardiography revealed an akinetic anterior wall. On day 4 following the first onset of pain, CMR was undertaken to assess anterior wall viability before invasive angiography. Hyperpolarized [1-^13^C]pyruvate imaging was also undertaken at this time. Late gadolinium enhancement imaging revealed transmural (75% to 100%, nonviable) infarction in the mid- and apical anterior walls, alongside the mid-anterolateral and mid-apical lateral walls (5 of 17 segments), with significant microvascular obstruction typical of acute infarction (Figure 1D). Hyperpolarized [1-^13^C]pyruvate imaging (Figure 1E) showed absent ^13^C-bicarbonate and [1-^13^C]lactate signals in the transmural infarction, but both ^13^C-bicarbonate and [1-^13^C]lactate signals were observed in the inferior lateral walls. Management options were discussed with the patient, and a conservative course of action was pursued in the first instance, with invasive angiography reserved for any recurrence of symptoms.

This is the first report to our knowledge of in vivo imaging of pathological metabolism in the human heart using hp-MRI. These 2 cases show that, whereas nonviable segments with transmural infarction show reduced PDH-mediated aerobic conversion to ^13^C-bicarbonate, viable segments following subendocardial infarction have preserved ^13^C-bicarbonate signal. This shows the difference in ongoing oxidative metabolism (the hallmark of viability) that exists between viable (hibernating) and nonviable myocardium. Further studies are now needed to investigate whether such a biomarker could be useful in stratifying those that would benefit from revascularization. Despite the presence of reduced PDH flux in the diabetic heart, previous work has demonstrated the potential for this technique to be applied in the diabetic heart (7). Because the subject in case 1 also had type 2 diabetes, this work further emphasizes the ability of hp-MRI to image metabolism in the diabetic heart.

In addition, we have shown that following hyperpolarized [1-^13^C]pyruvate injection, [1-^13^C]lactate signals were absent in the nonviable sections, but were seen in the viable recently infarcted segment in case 1 and in the remote myocardium in case 2. Although this possibly represents residual ischemia in the infarcted segment in case 1, this may also be explained by inflammatory changes. The origin of these [1-^13^C]lactate signals requires further clarification.

These results demonstrate the emerging potential for hyperpolarized imaging in ischemic heart disease. The detection of downstream conversion to either bicarbonate or lactate after hyperpolarized [1-^13^C]pyruvate injection has the potential to characterize the metabolic state of viable myocardium noninvasively. Because this can be achieved in a single 90-s scan, and in the absence of ionizing radiation, this is an exciting prospect for future cardiovascular research.
